# Targeting virulence regulation to disarm *Acinetobacter baumannii* pathogenesis

**DOI:** 10.1080/21505594.2022.2135273

**Published:** 2022-10-19

**Authors:** Vincent Trebosc, Valentina Lucchini, Mohit Narwal, Basil Wicki, Sarah Gartenmann, Birgit Schellhorn, Julian Schill, Marilyne Bourotte, Daniel Frey, Jürgen Grünberg, Andrej Trauner, Livia Ferrari, Antonio Felici, Olivia L. Champion, Marc Gitzinger, Sergio Lociuro, Richard A. Kammerer, Christian Kemmer, Michel Pieren

**Affiliations:** a BioVersys AG, Basel, Switzerland; b Biozentrum, University of Basel, Basel, Switzerland; c BioVersys SAS, Lille, France; d Laboratory of Biomolecular Research, Division of Biology and Chemistry, Paul Scherrer Institute, Villigen, Switzerland; e Center for Radiopharmaceutical Sciences ETH-PSI-USZ, Paul Scherrer Institute, Villigen, Switzerland; f Microbiology Discovery, Aptuit Srl, an Evotec Company, Verona, Italy; g Biosystems Technology Ltd, Exeter, UK

**Keywords:** OmpR, *A. baumannii*, virulence, drug discovery

## Abstract

The development of anti-virulence drug therapy against *Acinetobacter baumannii* infections would provide an alternative to traditional antibacterial therapy that are increasingly failing. Here, we demonstrate that the OmpR transcriptional regulator plays a pivotal role in the pathogenesis of diverse *A. baumannii* clinical strains in multiple murine and *G. mellonella* invertebrate infection models. We identified OmpR-regulated genes using RNA sequencing and further validated two genes whose expression can be used as robust biomarker to quantify OmpR inhibition in *A. baumannii*. Moreover, the determination of the structure of the OmpR DNA binding domain of *A. baumannii* and the development of *in vitro* protein-DNA binding assays enabled the identification of an OmpR small molecule inhibitor. We conclude that OmpR is a valid and unexplored target to fight *A. baumannii* infections and we believe that the described platform combining *in silico* methods, *in vitro* OmpR inhibitory assays and *in vivo G. mellonella* surrogate infection model will facilitate future drug discovery programs.

## Introduction

The rapid development of antimicrobial resistance is an increasing serious issue and a global threat to public health [1,2]. The need for new antibacterial therapeutics is especially critical for drug-resistance Gram-negative bacteria, such as carbapenem-resistant *Acinetobacter baumannii* (CRAB) for which new approaches are urgently required [3]. Several European countries reported an endemic CRAB situation in 2019 [4] and the situation is also alarming in China and in U.S., [5,6]. Alternative approaches to traditional antimicrobials consist in targeting bacterial resistance or virulence mechanisms to disarm the pathogens [7–9]. The pathogenicity of *A. baumannii* is not fully understood but several virulence factors have been identified as part of its disease-causing ability, such as its ability to survive in adverse environmental conditions and to escape the host immune system [10,11].

Two-component systems (TCSs) play a crucial role in sensing and responding to environmental changes by adapting to diverse ecological niches, even under unfavourable conditions [12]. Typically, TCSs consist of an histidine kinase (HK) that senses specific external stimuli, and a response regulator (RR) that mediates the cellular response through differential expression of target genes. TCSs are often involved in the regulation of virulence genes expression [13], indicating that TCSs could serve as potential novel drug target in the development of anti-virulence strategies [14]. OmpR/EnvZ is a well-studied TCS in *Enterobacteriaceae*, where OmpR is the RR and EnvZ is the HK sensor [15–17]. Under osmolarity changes in specific cellular niches, OmpR regulates the expression of about 100 genes, including outer membrane proteins and virulence factors, such as fimbriae and pili [18,19]. In contrast, the role of OmpR is poorly explored in *A. baumannii* [20].

The aim of our study was to gain insight in the role of OmpR in *A. baumannii* pathogenesis to enable the development of OmpR inhibitors altering *A. baumannii* virulence. We demonstrated that OmpR is a key regulator of *A. baumannii* virulence using different infection models and diverse clinical strains. We further identified OmpR-regulated genes that can be used as biomarker of OmpR inhibition in whole cell assay. In addition, we solved the structure of the C-terminal domain of OmpR and developed a protein-DNA *in vitro* binding assay leading to the identification of an OmpR small molecule inhibitor derived from a virtual screening.

## Results

### OmpR is required for A. baumannii virulence in diverse mouse infection models

**Figure 1.**
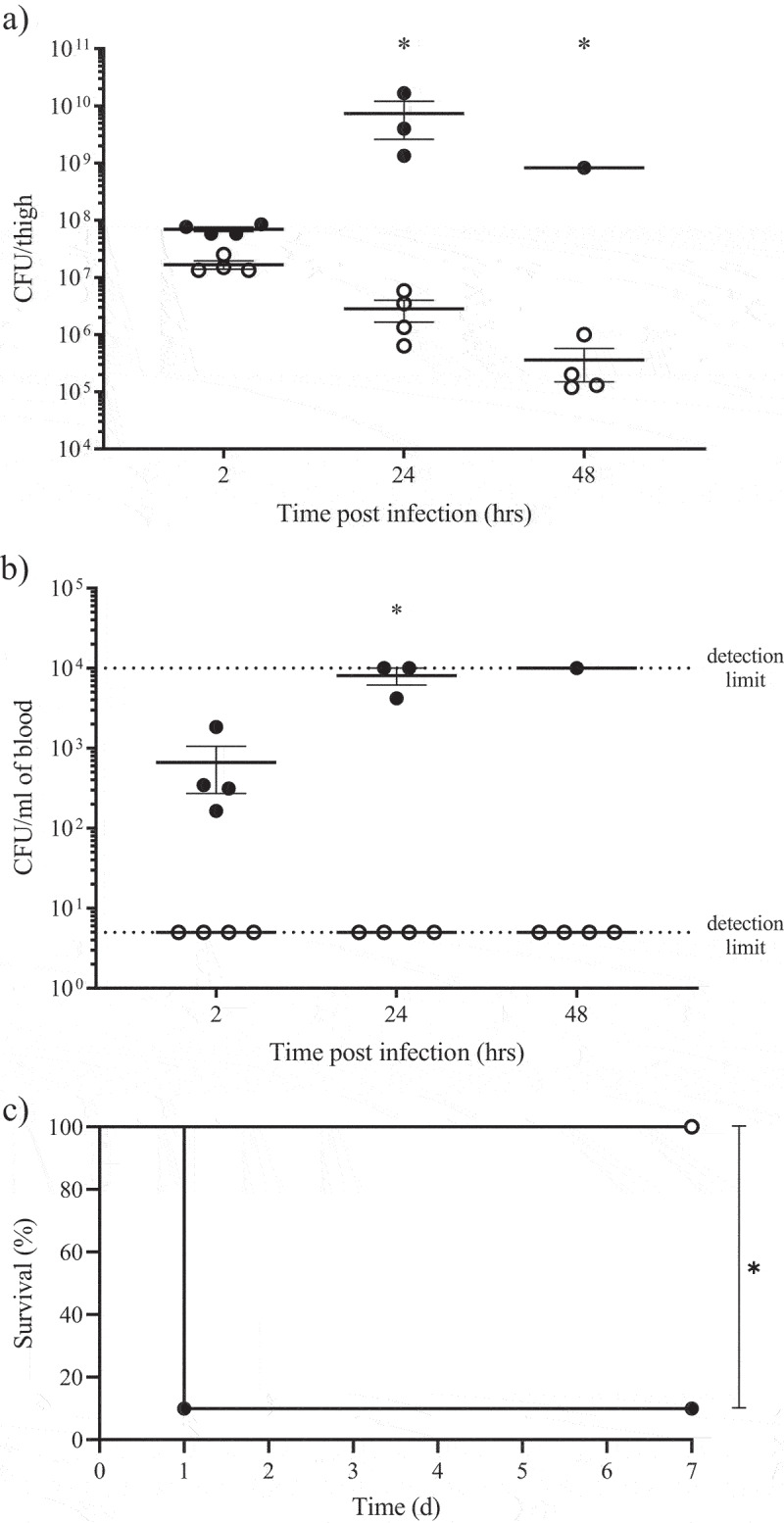
Role of OmpR in *A. baumannii* virulence assessed in thigh and septicaemia mouse infection models.

It has been recently suggested that OmpR plays a role in *A. baumannii* pathogenesis in the *G. mellonella* invertebrate infection model using the AB5075 strain [20]. We first evaluated if the attenuated virulence observed in *G. mellonella* correlates with the bacterial pathogenesis in vertebrate infection models. Neutropenic mouse thigh infection with the AB5075 wildtype strain resulted in a 2-log_10_ increase in thigh bacterial titre and a rapid dissemination of the infection in the blood, leading to the mortality of 1 and 3 animals at 24 and 48 hours, respectively (Figure 1(a,b)). In contrast, a 1 and 2-log_10_ reduction in thigh bacterial titer at 24 and 48 hours, respectively, no blood dissemination and no mortality was observed for the AB5075 Δ*ompR* mutant (Figure 1(a,b)). These data indicate that OmpR is required for AB5075 to establish a robust infection in thigh infection model. Since *A. baumannii* is a threat pathogen causing sepsis, we next evaluated the role of OmpR in an immunocompetent septicaemia mouse model with the unrelated and highly virulent *A. baumannii* strain HUMC1 [21]. Infection with the wildtype HUMC1 strain led to 90% mortality at day one while the HUMC1 Δ*ompR* mutant did not induce any mortality after 7 days (Figure 1(c)). Overall, *ompR* deletion led to strongly attenuated *A. baumannii* virulence protecting mice from bacteria-mediated killing and demonstrating the importance of OmpR in *A. baumannii* pathogenesis.

A) and B) Neutropenic CD-1 male mice (12 per group) were infected using thigh injection of 10^7^ cfu of AB5075 wildtype (black) or AB5075 Δ*ompR* mutant (white) and 4 animals per group were sacrificed at 2, 24 and 48 hours post infection for bacterial titers determination in A) thigh and B) blood. Mortality was observed in the wildtype group at 24 and 48 hours enabling bacterial titers analysis on only 3 and 1 animal, respectively. Unpaired *t*-test, *P-value < 0.05. C) Immunocompetent C57BL6J male mice (10 per group) were infected with intravenous injection of 5 × 10^7^ cfu of HUMC1 wildtype (black) or HUMC1 Δ*ompR* mutant (white) and mice survival was monitored for 7 days. Log-rank (Mantel-Cox) test, *P-value < 0.05.

### G. mellonella infection model can be used as surrogate of mouse model to characterize OmpR-mediated A. baumannii virulence

**Figure 2.**
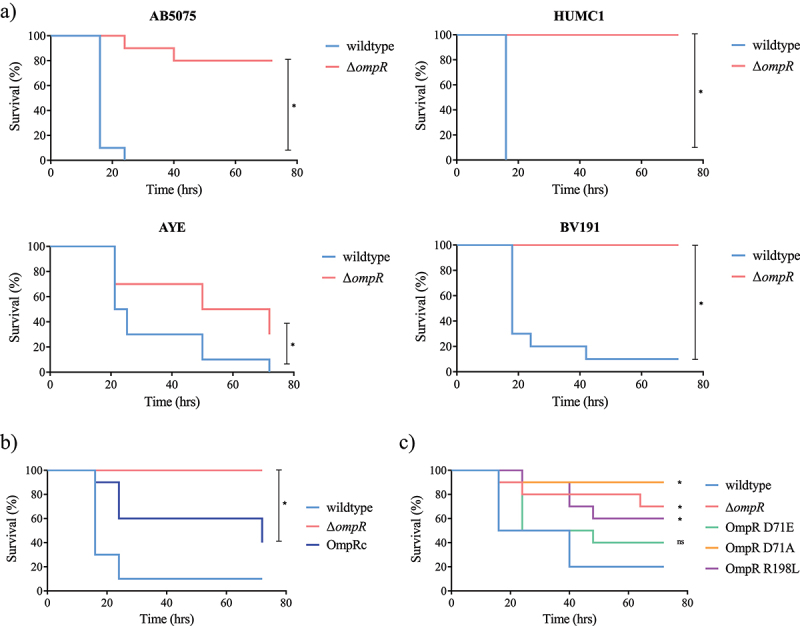
Role of OmpR in *A. baumannii* virulence assessed in *G. mellonella* infection model.

We implemented the *G. mellonella* invertebrate infection model as a more ethical and cost-effective surrogate to mouse infection models [22,23] to further evaluate the role of OmpR in *A. baumannii* pathogenesis using additional clinical strains. Deletion of *ompR* led to attenuated virulence in the four diverse clinical strains tested in *G. mellonella* model and protected the larvae from bacteria-mediated killing, suggesting that the role of OmpR in bacterial virulence is conserved in *A. baumannii* (Figure 2(a)). Importantly, chromosomal complementation of the Δ*ompR* mutant significantly increased the virulence of *A. baumannii* AB5075 in the *G. mellonella* model, confirming that the reduced virulence of the Δ*ompR* mutant is due to the loss of OmpR (Figure 2(b)). To further dissect the role of OmpR in *A. baumannii* virulence we used mutant strains expressing chromosomal OmpR variants having different activation states or DNA binding ability. Based on previous work performed on OmpR *E. coli*, [24] the aspartic acid at position 71 of *A. baumannii* OmpR, which in *E. coli* corresponds to the aspartic acid residue at position 55 that is phosphorylated upon EnvZ-mediated OmpR activation, was substituted by either an alanine (D71A) to abolish OmpR activation or by a glutamic acid (D71E) to induce OmpR-activated conformation. The inability of OmpR D71A and D71E mutants to be phosphorylated was confirmed *in vitro* (Supplementary Figure S1). In addition, the arginine at position 198 (R182 in *E. coli*) was substituted by a leucine (R198L) to prevent OmpR binding to DNA [25]. Infection of *G. mellonella* larvae with the WT and OmpR D71E chromosomal mutant led to rapid larvae killing with 50% mortality observed at 24 h that was further increasing until 72 h post infection (Figure 2(c)). In contrast, larvae infected with the Δ*ompR* mutant and the OmpR D71A and OmpR R198L chromosomal mutants that prevent phosphorylation or DNA binding, respectively, showed reduced mortality (10–40%) at 72 h. Overall, the chromosomal mutants impaired for OmpR phosphorylation (D71A) or for DNA binding (R198L) were attenuated similarly to the Δ*ompR* mutant, indicating that both OmpR characteristics are required for *A. baumannii* virulence.

A) Groups of 10 larvae were infected with 10^5^ cfu of AB5075, HUMC1, AYE or BV191 wildtype strains (blue) and their isogenic Δ*ompR* mutants (red). B) Groups of 10 larvae were infected with 10^4^ cfu of AB5075 wildtype (light blue), AB5075 Δ*ompR* (red) and OmpR complemented (OmpRc, dark blue) strains. C) Groups of 10 larvae were infected with 10^4^ cfu of AB5075 wildtype (light blue), AB5075 Δ*ompR* (red), AB5075 OmpR D71E (green), AB5075 OmpR D71A (orange) and AB5075 OmpR R198L (purple) mutants. Larvae survival was monitored over 72 hours. Log-rank (Mantel-Cox) test compared to wildtype, *P-value < 0.05.

**Figure 3.**
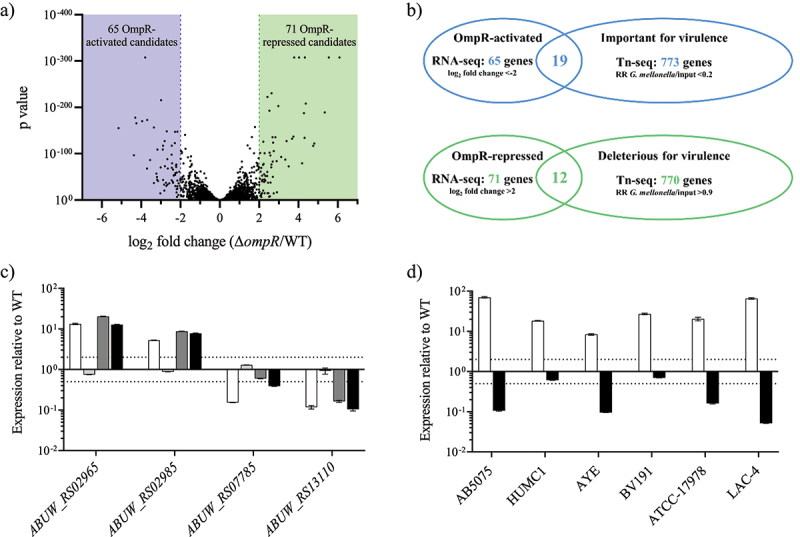
Identification and confirmation of OmpR-regulated genes.

A) RNA-seq results representation of differentially expressed genes between AB5075 wildtype and its Δ*ompR* mutants. Sixty-five OmpR-activated candidate genes (<-2 log_2_ fold change, blue zone) and 71 OmpR-repressed candidate genes (>2 log_2_ fold change, green zone) were identified. B) The RNA-seq results were combined with a Tn-seq dataset conducted to identify *A. baumannii* genes that are important or deleterious for *A. baumannii* virulence [26]. Nineteen OmpR-activated candidate genes were important for *A. baumannii* virulence whereas 12 OmpR-repressed candidate genes were deleterious for *A. baumannii* virulence. RR: read ratio. C) The transcript levels were determined in *A. baumannii* AB5075 wildtype, AB5075 Δ*ompR* (white), AB5075 OmpR D71E (light grey), AB5075 OmpR D71A (dark grey) and AB5075 OmpR R198L (black) mutants by quantitative real-time PCR and the expression level was normalized to the expression of the AB5075 wildtype strain (means ± SEM of two technical replicates). D) The transcript levels of *ABUW_RS02965* (white) and *ABUW_RS13110* (black) were determined in *A. baumannii* Δ*ompR* mutants and normalized to the transcript levels of their respective wildtype strains (means ± SEM of two technical replicates). Horizontal dotted lines depict a 2-fold up- or downregulation.

### Identification of OmpR-regulated genes as biomarkers of OmpR inactivation in A. baumannii

By using different vertebrate and non-vertebrate *in vivo* models we have shown that OmpR is required for *A. baumannii* pathogenesis, suggesting that OmpR inhibition may be an alternative approach to block bacterial virulence and potentially treat *A. baumannii* infections. However, *in vitro* assays to estimate the OmpR activity are required for the identification of inhibitors. In this regard, we aimed at identifying OmpR-regulated genes that could be used as biomarkers for OmpR inhibition in *A. baumannii*.

A comparative RNA sequencing (RNA-seq) study was carried out with the AB5075 strain and its isogenic ∆*ompR* mutant, which led to the identification of 65 genes that were downregulated in Δ*ompR* (<-2 log_2_ fold change, Δ*ompR*/WT) and 71 genes that were upregulated in Δ*ompR* (>2 log_2_ fold change, Δ*ompR*/WT) (Figure 3(a) and Supplementary Table S1). The identified downregulated genes correspond to OmpR-activated candidate biomarkers while the upregulated genes correspond to OmpR-repressed candidate biomarkers. To identify OmpR biomarkers that are relevant for *A. baumannii* pathogenesis, we combined our RNA-seq data with the data from a transposon insertion sequencing (Tn-seq) that was conducted to identify AB5075 virulence determinant in the *G. mellonella* model [26]. Considering that OmpR is essential for virulence in *G. mellonella*, we hypothesized that genes that are downregulated in the Δ*ompR* mutant (RNA-seq) should match with genes that are important for *G. mellonella* infection (underrepresented in Tn-seq). Likewise, genes overexpressed in the Δ*ompR* mutant (RNA-seq) may match with genes that are deleterious for *G. mellonella* infection (overrepresented in Tn-seq). This analysis identified 19 OmpR-activated candidate genes and 12 OmpR-repressed candidate genes that may be relevant for *A. baumannii* pathogenesis (Figure 3(b) and Supplementary Table S2).

We selected four of those candidate marker genes (2 up- and 2 downregulated) for confirmation using qRT-PCR on the chromosomal mutants expressing different OmpR variants. The upregulated genes *ABUW_RS02965* and *ABUW_RS02985* and the downregulated genes *ABUW_RS07785* and *ABUW_RS13110* showed the expected expression pattern in the strains with active (WT and OmpR D71E) and inactive (Δ*ompR*, OmpR D71A and OmpR R198 L) OmpR (Figure 3(c)). These results confirmed that the expression of these genes is regulated by OmpR and that both OmpR activation and DNA binding properties are important for correct regulation. Three of the confirmed OmpR-regulated genes, namely *ABUW_RS02965*, *ABUW_RS02985* and *ABUW_RS07785*, encode small hypothetical proteins (<150 amino acids) with one of them (*ABUW_RS02965*) belonging to the RcnB protein family involved in metal ion homoeostasis [27]. The last OmpR-regulated gene (*ABUW_RS13110*) encodes for an amino acid efflux protein from the LysE transporter family named MatE. Interestingly, several Δ*ompR* upregulated genes encode for small proteins similar to *ABUW_RS02965* and *ABUW_RS02985*, and their OmpR mediated expression regulation was as well confirmed (Table S2 and Figure S2A). Finally, OmpR expression regulation of the most upregulated gene (*ABUW_RS02965*) was conserved in all the clinical strains tested while OmpR expression regulation of the most downregulated gene (*ABUW_RS13110*) was conserved in 4 of the 6 clinical strains tested (Figure 3(d)). Moreover, the expression of *ABUW_RS02965* was partially restored (20- to 4-fold) while the expression of *ompR* and *ABUW_RS13110* was fully restored in the OmpR chromosomal complemented strain, confirming that the up- and downregulation observed in the Δ*ompR* mutant are due to the loss of OmpR (Supplementary Figure S2B). Together, our data demonstrate that the expression of the genes *ABUW_RS02965* and *ABUW_RS13110* is repressed and activated by OmpR, respectively, and that monitoring of their expression may serve as biomarker of OmpR inhibition in most *A. baumannii* strains.

### Development of an OmpR DNA binding assay and identification of OmpR DNA binding sites in A. baumannii.

**Figure 4.**
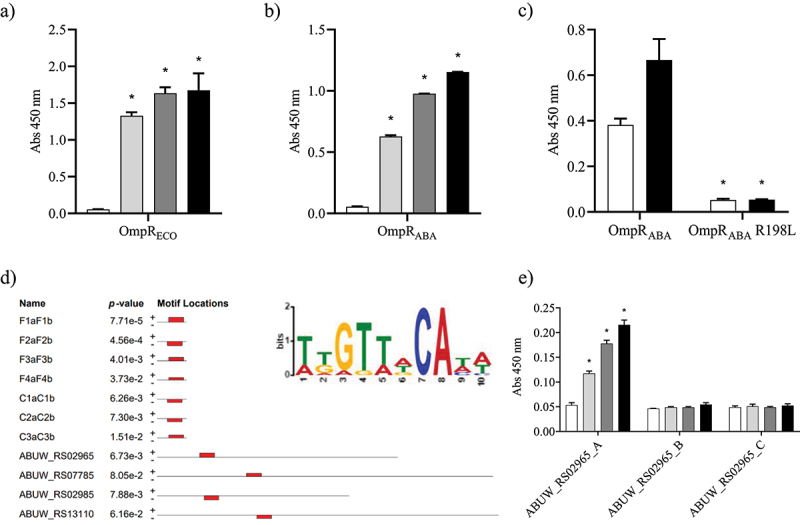
Study of OmpR protein-DNA binding using D-ELISA and *in silico* analysis.

We aimed to develop an *in vitro* assay enabling the quantification of OmpR DNA binding to further expand the toolbox for OmpR inhibitor discovery. The DNA-binding motif of OmpR has been described in *E. coli* (Supplementary Figure S3) [18]. More specifically, the binding between OmpR and the different binding sites present upstream of the OmpR-regulated genes *ompC* and *ompF* has been extensively studied in *E. coli* [15,28,29]. We used this knowledge to develop a DNA binding enzyme-linked immunosorbent (D-ELISA) sandwich assay where a biotinylated oligonucleotide encoding the OmpR binding site is attached to a streptavidin coated plate and serves as a bait for the transcription factor (Supplementary Figure S3). We confirmed the functionality of the D-ELISA assay using OmpR_ECO_ and its cognate DNA binding site F1aF1b (Figure 4(a)). Furthermore, we showed that OmpR_ABA_, which share 69% amino acid identity with OmpR_ECO_, is also binding to the *E. coli* F1aF1b binding site (Figure 4(b)). Moreover, we showed that OmpR_ABA_ phosphorylation increased DNA binding whereas the OmpR_ABA_ R198 L mutant was unable to bind DNA, suggesting that the D-ELISA assay allows quantification of OmpR DNA binding (Figure 4(c)).

OmpR binding to the F1aF1b oligonucleotide (2 µg/mL) was assessed with 3.1 µg/mL (light grey), 6.2 µg/mL (dark grey) and 12.5 µg/mL (black) of the OmpR_ECO_ (A) and OmpR_ABA_ (B) proteins. A negative control without protein was included (white). Unpaired t-test compared to control, *P-value < 0.05. C) The binding of OmpR_ABA_ and OmpR_ABA_ R198L mutant (6 µg/mL) to the F1aF1b oligonucleotide (2 µg/mL) was tested under unphosphorylated (white) and phosphorylated (black) conditions. Unpaired t-test compared to wildtype, *P-value < 0.05. D) A 10 nucleotide motif (highlighted in red) common to all known OmpR_ECO_ DNA binding sites upstream of *ompF* (F1aF1b, F2aF2b, F3aF3b and F4aF4b) and *ompC* (C1aC1b, C2aC2b and C3aC3b) was identified in the intergenic region upstream of the 4 OmpR_ABA_ regulated genes using the MEME suite [30]. The weblogo of the sequence motif is depicted in the upper right corner. E) OmpR_ABA_ binding to the ABUW_RS02965_A, B and C oligonucleotides (2 µg/mL) was assessed with 1.5 µg/mL (light grey), 3 µg/mL (dark grey) and 6 µg/mL (black) of proteins. A negative control without protein was included (white). Only the ABUW_RS02965_A encodes the identified OmpR DNA binding motif (Supplementary Figure S4). Unpaired t-test compared to control, *P-value < 0.05. The data represent the mean ± SD of at least two replicates.

As OmpR_ABA_ binds to cognate OmpR_ECO_ binding sites, we next looked whether the consensus OmpR_ECO_ DNA binding motif can be found upstream of the OmpR_ABA_-regulated genes that we have identified previously. A 10-nucleotide motif present in all the OmpR_ECO_ DNA binding sites upstream of *ompF* and *ompC* was identified upstream of the four OmpR_ABA_-regulated genes studied in this work (Figure 4(d) and Supplementary Figure S4). This motif corresponds to the previously identified OmpR_ECO_ DNA binding motif shifted by one nucleotide (Supplementary Figure S3). We used the intergenic region upstream of the gene *ABUW_RS02965* to confirm OmpR_ABA_ binding using D-ELISA assay. Three different oligonucleotides (ABUW_RS02965_A, B and C) that span the entire intergenic region were designed and only the oligonucleotide ABUW_RS02965_A included the identified OmpR DNA binding motif (Supplementary Figure S4). OmpR_ABA_ showed dose-dependent binding only to the oligonucleotide encoding the identified OmpR DNA binding site, confirming that OmpR_ABA_ specifically binds to the identified motif (Figure 4(e)). Together, our data suggest that the expression of the four marker genes is directly regulated by OmpR in *A. baumannii*.

### Structure of the DNA-binding domain of OmpR from A. baumannii

**Figure 5.**
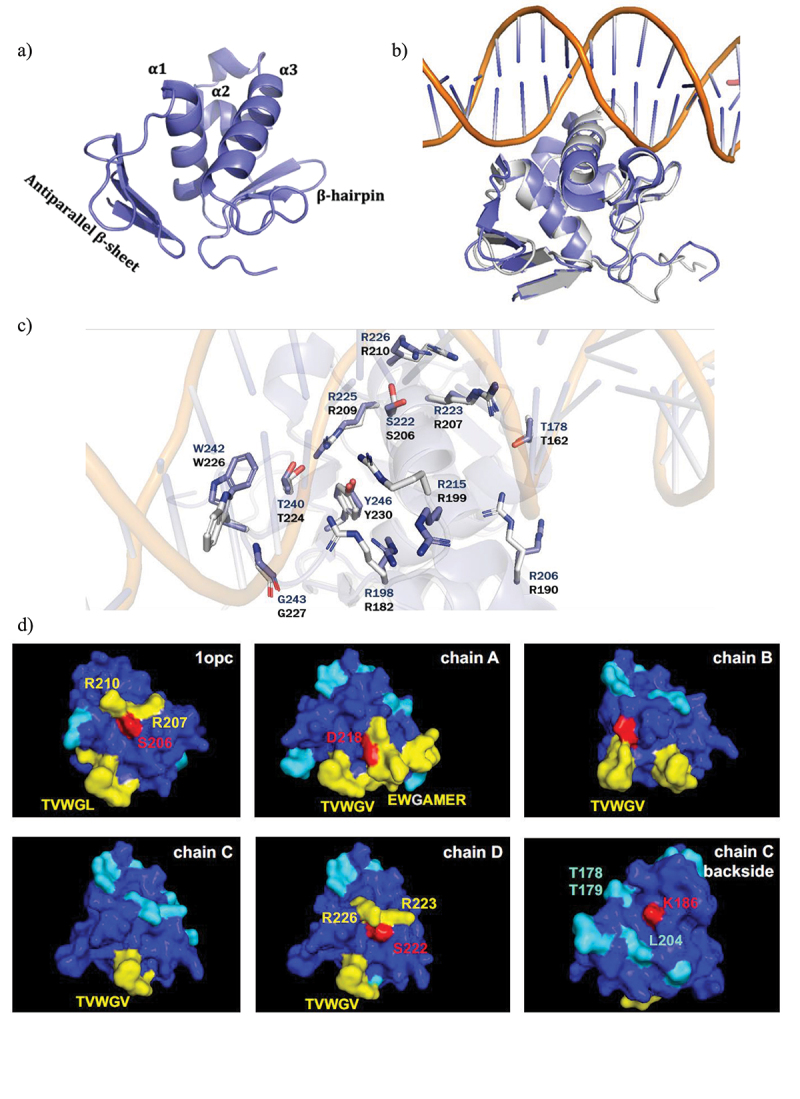
Structure of the DNA-binding domain of *A. baumannii* OmpR and computational hotspots analysis.

To enable the discovery of a small molecule inhibitor of OmpR, we solved the crystal structure of OmpR_ABA_ DNA binding domain (DBD) spanning the C-terminal residues 148 to 254 at a resolution of 2.2 Å. The analysis of the crystal structure showed that OmpR_ABA_ DBD consists of a three-stranded antiparallel β-sheet (β1, V153-F156; β2, W159-D162; β3, R166-T169) followed by three α-helices (α1, T179-E190; α2, R198-R206; α3, R215-I228) and ending with a β-hairpin (β4, I238-V241; β5, V244-F248) (Figure 5(a)). The arrangement of the β-sheets and α-helices represents the winged helix-turn-helix (wHTH) fold characteristic of DNA-binding proteins of the OmpR superfamily [31]. Notably, the OmpR_ABA_ DBD superimpose very well (root mean square deviation of 0.543 Å) with the OmpR_ECO_ DBD bound to DNA whose crystal structure has recently been determined (PDB entry: 6LXN) (Figure 5(b)) [32]. The sequence identity between OmpR_ABA_ DBD and OmpR_ECO_ DBD is 69% and all the amino acid residues of OmpR_ECO_ that are involved in the DNA binding were conserved in OmpR_ABA_ (Figure 5(c)). These observations suggest a very similar DNA-binding mode for both proteins, which is in line with OmpR_ABA_ binding to OmpR_ECO_ cognate binding site.

A) Overview of the crystal structure of the DNA-binding domain (DBD) of *A. baumannii* OmpR. B) Superposed crystal structure of OmpR DBD from *A. baumannii* (blue) on DNA bound structure of OmpR from *E. coli* (grey). C) Amino acids (blue colored for OmpR_ABA_ and grey colored for OmpR_ECO_) that are involved in making H-bond and salt bridges with DNA are shown. D) The 3D structures of OmpR_ECO_ (1opc) and OmpR_ABA_ DBD (chain A, B, C and D) are represented and colored based on temperature scale from blue (low ligand interaction probability) to red (high ligand interaction probability). Individual residues with high predicted ligand-binding potential are highlighted. The interaction site is around the conserved residues TVWG(V/L). Hotspot 1 in OmpR_ABA_-DBD chain A contains D218, as anchor residue for ligand binding. Hotspot 2 in OmpR_ABA_-DBD chain D contains residues S222, R223 and R226 (S206, R207 and R210 in OmpR_ECO_ DBD). Hotspot 3 in OmpR_ABA_-DBD chain C contains residues K186, L204, and T178/T179.

### Identification of OmpR inhibitor tool compound using in silico screening approach.

**Figure 6.**
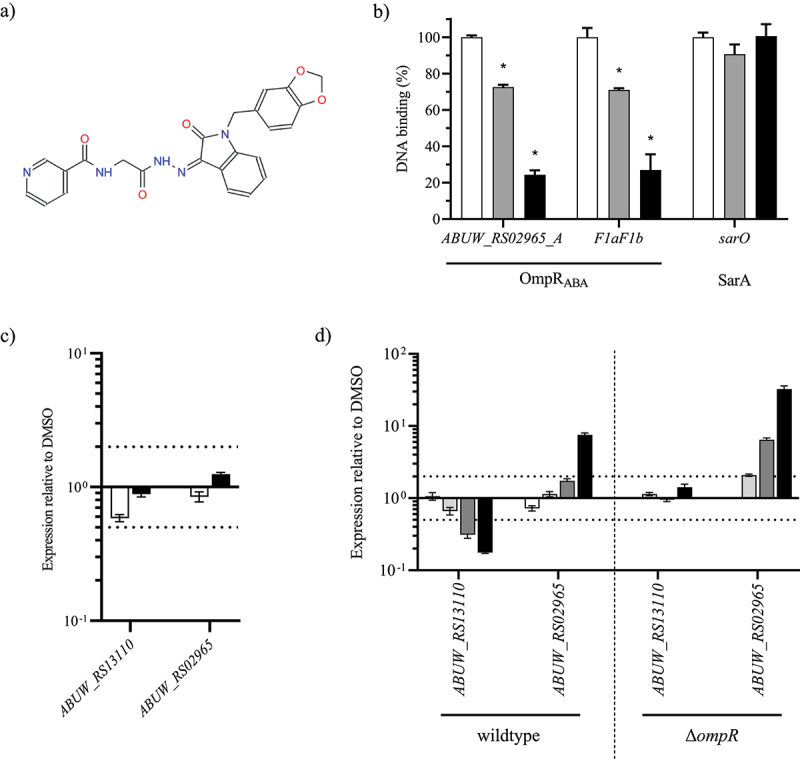
OmpR inhibitory activity of VSIS_039.

We finally aimed at discovering small drug inhibitor of OmpR using the different tools developed in this study. A hotspot analysis was used to identify potential protein-ligand binding sites on the solvent-accessible surface of protein structures 1OPC and of OmpR_ABA_ DBD [33]. Three hotspots embedding highly predicted anchor residues as potential ligand binding pockets in the identification of DNA-inhibitors (Figure 5(d)) were identified. The most likely interaction site (hotspot 1) in all structures is around conserved residues T240VWG(V/L)244 in OmpR_ABA_ DBD. All structures possess this feature, most pronounced in chain A. According to RCSB-PDB annotation for 1OPC, this stretch of residues might be the RNA polymerase interaction site. The predicted residues flank a pocket that may serve as a ligand binding site. Residue D218 in chain A may serve as an anchor point. OmpR_ABA_ DBD chain D features a pronounced predicted secondary site (hotspot 2) around residues S222, R223 and R226 (S206, R207, R210 in 1OPC). The “backside” of OmpR_ABA_ DBD chain C features a third potential ligand binding pocket flanked by residues K186 (potential anchor residue), L204 and T178/T179. The structure-based pharmacophore model [34] was used for a virtual screening to search for compounds against hotspots 1 and 2 that can block OmpR DNA binding, abrogating both its repression and activation functions. More than 40 structural distinct inhibitors were retrieved as final top-scoring candidates from different commercially available chemical suppliers. We used our developed D-ELISA to evaluate the inhibitory effect of the virtual hits on OmpR_ABA_ DNA binding. We only observed a dose-dependent inhibition of OmpR_ABA_ DNA binding for the compound VSIS_039 (STL300125, Vitas-M Chemical Limited), which was identified to bind to the hotspot 2 druggable pocket of OmpR (Figure 6(a,b)). Moreover, VSIS_039 did not inhibit the binding of an unrelated transcriptional regulator (SarA from *S. aureus*) to its cognate DNA binding site [35], indicating that *in vitro* VSIS_039 mediated DNA binding inhibition is specific to OmpR. However, VSIS_039 did not affect the expression of the OmpR_ABA_ marker genes *ABUW_RS02965* and *ABUW_RS13110* when tested at up to 100 µM in whole cell-based assay (<2-log fold change) (Figure 5(c)). These results suggest that VSIS_039 is unable to efficiently enter in *A. baumannii* or is prone to rapid efflux in *A. baumannii*. We constructed an *A. baumannii* AYE mutant deleted for the three conserved and main resistance-nodulation-division (RND) efflux pumps of *A. baumannii*, namely *adeABC*, *adeFGH* and *adeIJK*. By using the Δ*ompR* mutant of the efflux depleted strain, we confirmed that OmpR-regulation of *ABUW_RS02965* and *ABUW_RS13110* is not affected by the deletion of the efflux pumps (Supplementary Figure S5A), enabling to test the OmpR inhibition activity of VSIS_039 on the efflux depleted mutant. VSIS_039 demonstrated a dose dependent upregulation of *ABUW_RS02965* and downregulation of *ABUW_RS13110* in the AYE efflux depleted strain while it did not have antibiotic activity (MIC >200 µM, HepG2 LD_50_ >100 µM) (Figure 5(d)). The compound was further tested on the Δ*ompR* mutant of the efflux depleted strain to control that the inhibitor is acting through OmpR. No effect was observed on the expression of *ABUW_RS13110*, suggesting that the compound is targeting OmpR, whereas the second biomarker gene (*ABUW_RS02965*) was upregulated as in the wildtype strain, suggesting that the compound may also have additional targets that are involved in *ABUW_RS02965* regulation. Finally, using the intermediate efflux mutants, we showed that deletion of the *adeABC* efflux pump is not sufficient for VSIS_039 activity, which was observed in the Δ*adeABC* Δ*adeIJK* double deletion mutant and did not further increase in the Δ*adeABC* Δ*adeIJK* Δ*adeFGH* triple deletion mutant (Supplementary Figure S5B). Together with the D-ELISA results, the biomarker expression studies suggest that VSIS_039 alters the activity of *A. baumannii* OmpR through the inhibition of OmpR DNA binding and that VSIS_039 is a substrate of the *A. baumannii* RND efflux pumps AdeIJK, preventing its use on wildtype strains. The impact of VSIS_039 on *A. baumannii* virulence could not be evaluated as the AYE efflux depleted mutant was avirulent in *G. mellonella* model, which is consistent with efflux pumps playing an important role in *A. baumannii* and other Gram-negative pathogenesis [26,36–39]. Alternatively, Tipton et *al*. reported the role of OmpR in *A. baumannii* motility [20]. However, motility was not affected by the deletion of *ompR* in the AYE efflux depleted strain (Supplementary Figure S6), preventing the use of this phenotype to assess the activity of VSIS_039.

A) Structure of the compound VSIS_039 (STL300125, Vitas-M Chemical Limited). B) OmpR_ABA_ DNA inhibition was assessed by D-ELISA using 2 µg/mL biotinylated ABUW_RS02965_A and F1aF1b oligos and 6 µg/mL of OmpR_ABA_ in the presence of DMSO (white), 10 µM (grey) and 20 µM (black) of VSIS_039 compound (mean ± SD of 2 technical replicates). As negative control, VSIS_039 was also tested on SarA and its cognate DNA binding site using 10 µg/mL of the SarA protein and 2 µg/mL of the biotinylated *sar0* oligos. Unpaired t-test compared to DMSO, *P-value < 0.05. C) The transcript levels of *ABUW_RS02965* and *ABUW_RS13110* were determined in the *A. baumannii* AYE wildtype strain in the presence of VSIS_039 at 33.3 μM (white) and 100 μM (black). The transcripts levels were normalized to those of the DMSO control (mean ± SEM of 2 technical replicates). D) The transcript levels of *ABUW_RS02965* and *ABUW_RS13110* were determined in the *A. baumannii* AYE efflux depleted (Δ*adeABC* Δ*adeFGH* Δ*adeIJK*) wildtype and Δ*ompR* strains in the presence of VSIS_039 at 10 μM (white), 20 μM (light grey), 40 μM (dark grey) and 80 μM (black). The transcripts levels were normalized to those of the DMSO control (mean ± SEM of 2 technical replicates). Horizontal dotted lines depict a 2-fold up- or downregulation.

## Discussion

Target validation is often the first step of drug discovery and it is particularly relevant for adjuvant drugs, such as anti-virulence drugs, targeting non-essential (at least *in vitro*) pathways, which may be less conserved than essential pathways that are targeted by antibiotics [40–42]. In this study we demonstrated that OmpR is essential for different *A. baumannii* clinical isolates to establish a robust infection in thigh and septicaemia mouse models as well as in *G. mellonella* invertebrate model. These results are consistent with the findings of a previous study, suggesting that OmpR plays a role in AB5075 *A. baumannii* virulence in *G. mellonella* [20]. Interestingly, the results from two independent transposon mutagenesis studies on *A. baumannii* ATCC 17978 [43] and AB5075 [26] suggest that OmpR is important for growth in rich media while another transposon mutagenesis study on *A. baumannii* 307–0294 [44] identified EnvZ, the sensor kinase of the OmpR/EnvZ TCS, as an *in vivo* essential gene. In addition, an *in silico* subtractive genomic study identified EnvZ as essential for the viability of *A. baumannii* [45]. Together, these findings support that the OmpR/EnvZ TCS is a valid and unexploited anti-virulence drug target in *A. baumannii*.

The role of OmpR in *A. baumannii* pathogenesis echoes the important role of OmpR in the pathogenesis of adherent-invasive and uropathogenic *E. coli* [46,47]. OmpR-mediated adaptation to osmotic stress via differential expression of outer membrane proteins is required for uropathogenic *E. coli* virulence. Similarly, OmpR was shown to play an important role in *A. baumannii* osmotic stress response but no OmpR-regulated genes could be identified [20]. We identified 65 OmpR-downregulated candidate genes and 71 OmpR-upregulated candidate genes in *A. baumannii* and confirmed proper OmpR regulation of 10 of them. Interestingly, several OmpR-regulated genes encoding unknown proteins were suggested to be involved in *A. baumannii* virulence in *G. mellonella* [25]. Therefore, the mechanism of OmpR-mediated virulence in *A. baumannii* may be uncovered by understanding the function of those unknown proteins. However, additional work is required to first confirm the involvement of those unknow proteins in *A. baumannii* pathogenesis and to then uncover their role. Ultimately, the OmpR-mediated repression of *ABUW_RS02965* and the OmpR-mediated activation of *ABUW_RS13110* was confirmed on strains expressing different OmpR-inactivated variants, on the OmpR complemented strain and on several clinical isolates. Moreover, the identification of OmpR DNA binding site upstream of these two genes suggest that they are directly regulated by OmpR. Together, our results demonstrate that the expression of *ABUW_RS02965* and *ABUW_RS13110* can be used as robust biomarker of *A. baumannii* OmpR inhibition, both at the phosphorylation and DNA-binding levels.

The determination of the protein structure of a drug target represents an important tool for drug development [48]. The resolution of the OmpR C-terminal domain of *A. baumannii* enabled the identification of VSIS_039, an OmpR small molecule inhibitor that interferes with OmpR DNA binding as demonstrated by D-ELISA and mis-regulation of the OmpR biomarkers. However, VSIS_039 did not show activity in *A. baumannii* wildtype strains due to efflux of the drug by the AdeIJK RND efflux pumps. Cell permeability and efflux pumps are major barriers for the development of anti-infectives against Gram-negative species [49,50]. In contrast to cell based *in vitro* screening, the main drawback of target based *in silico* and *in vitro* screening assays is that they do not account for molecule permeation and/or efflux. Although VSIS_039 permeation into *A. baumannii* seems not to be a bottleneck, further optimization of VSIS_039 is required to minimize efflux and gain whole cell OmpR inhibition activity against *A. baumannii*.

Interestingly, the predicted binding site of VSIS_039 to OmpR_ABA_ is conserved in OmpR from *Enterobacteriaceae* species. The structure conservation between *A. baumannii* and *Enterobacteriaceae* OmpR proteins opens the opportunity to develop an anti-virulence drug with a broader spectrum of activity. The development of anti-virulence drugs as an alternative to antimicrobials therapeutic is in constant progression with most approaches focusing on *Pseudomonas aeruginosa* and *Staphylococcus aureus* but less commonly on *A. baumannii* or broad spectrum [51]. Mannoside-mediated inhibition of FimH that compromises the ability of uropathogenic and adherent-invasive *E. coli* to colonize and invade the bladder and gut epithelium, respectively, is a notorious anti-virulence approach with drugs in clinical development to treat urinary tract infections and Crohn’s disease [52,53]. Interestingly, the expression of FimH has been shown to be positively regulated by OmpR, further highlighting the strong potential of an OmpR inhibitor as an anti-virulence drug [54].

In conclusion, this study validated OmpR as new anti-virulence target against *A. baumannii*. Precision therapeutics disarming pathogens without killing them may have the potential to treat infections while limiting the development of resistance [55,56]. However, these alternative approaches come with additional challenges during drug development, with one of them being the need of alternatives to traditional MIC to measure compound efficacy [42]. Here we developed and validated biochemical, cell based and *in vivo* assays that, together, can be used as a drug discovery platform for OmpR inhibitor development to treat *A. baumannii* infections. In addition, we confirmed that inhibition of OmpR DNA binding is sufficient to block downstream virulence pathways. With this information, it will be possible to setup a bacterial OmpR-dependent reporter screening assay to screen for molecule inhibitors.

## Material and methods

### Strains

The *A. baumannii* strains used is this study are summarized in (Supplementary Table S3). Bacteria were grown in Luria Bertani (LB) broth or on LB agar, incubation was done at 37°C with shaking (220 rpm) for liquid cultures and, when required, antibiotics were added as specified.

### Generation of mutants in A. baumannii clinical strains

Scarless deletions, allelic replacements and chromosomal insertion were performed using the pVT77 engineering platform and the two-step allelic exchange method previously described [40]. The lists of the engineered strains and oligonucleotides used in this study are available in (Supplementary Table S3 and Supplementary Table S4), respectively.

DNA fragments corresponding to 700-bp up- and downstream genomic regions of the gene to be deleted were amplified by PCR on the respective *A. baumannii* strain and cloned in the multiple cloning site (MCS) of pVT77. For the construction of the AB5075 OmpR chromosomal mutants, a 1.4-kb DNA fragment spanning *ompR* and centred on the codon to exchange was amplified from the *A. baumannii* AB5075 strain and cloned in the MCS of pVT77. Site directed PCR mutagenesis was performed on the resulting plasmids to introduce the OmpR D71A (GAC to GCC), OmpR D71E (GAC to GAG) and OmpR R198 L (CGC to CTA) mutations. For the construction of the AB5075 OmpR complemented strain, the DNA fragment containing the *ompR* gene and its promoter region was inserted between the *ponA* (*ABUW_RS01420*) and *rrm* (*ABUW_RS01415*) genes in the chromosome of the Δ*ompR* mutant. The DNA fragments spanning the *ponA* and *rrm* gene were first amplified with oVT425/426 and oVT427/428 and cloned (Gibson assembly) in the MCS (XhoI/SpeI) of the pVT77 plasmid. The DNA fragment containing *ompR* and its promoter was amplified using oVT630/631 and cloned (Gibson assembly) between *ponA* and *rrm* (EcoRI/SpeI), finally giving the plasmid ready for *ompR* chromosomal insertion using allelic exchange.

The cloned allelic exchange plasmids were conjugated in *A. baumannii*, and transconjugants were selected on LB agar plates containing 100 mg/L tellurite. The second recombination was selected on 200 mg/L 30-azido-30-deoxythymidine and the desired genetic modifications were confirmed by PCR and sequencing.

### Animals

All experiments involving animals were carried out in accordance with the European directive 2010/63/UE governing animal welfare and protection, which is acknowledged by the Italian Legislative Decree no 26/2014 and according to the company policy on the care and use of laboratories animals. All the studies were revised by the Animal Welfare Body and approved by Italian Ministry of Health (authorization n. 50 and 51/2014-PR)

Male CD-1 mice (Charles River Laboratories, Italy), 6 weeks old, were allowed 5 days for acclimation after the arrival. The mice were maintained on a 12-hour light cycle with ad libitum access to rodent feed (Altromin R, A. Rieper SpA, Italy) and filtered tap water. Animals were monitored during the entire period of the studies and clinical signs were recorded.

### Mouse infection models

For the neutropenic thigh infection model, male CD-1 mice of 18–20 days were made neutropenic by the administration of 150 and 100 mg kg^−1^ of cyclophosphamide, intraperitoneally, at 4 and 1 days before infection. Mice were briefly anaesthetized with isoflurane and infected intramuscularly in the thigh with 100 µl of 10^7^ cfu of *A. baumannii* AB5075 WT and *∆ompR* in exponential phase. Mice survival rate was recorded for 48 hours during the experimental phase. In addition, four animals/group were sacrificed under CO_2_ at 2-, 24- and 48-hours post infection and thighs were collected for cfu determination.

For the immunocompetent septicaemia infection model, C57BL6/J male mice of 7 weeks old were intravenously infected with 5 × 10^7^ cfu of *A. baumannii* HUMC1 WT and *∆ompR* strains in exponential phase. Mice survival rate was recorded until 7 days post infection.

Mouse infection experiments were performed at Aptuit (today Evotec), Verona, Italy.

### Galleria mellonella infection model

Ten *G. mellonella* larvae (TruLarv™, Bio Systems Technology) per group were infected using a 10-μl injection in the right second proleg with mid-log phase (OD600 = 0.5) growing bacteria resuspended in phosphate-buffered saline (PBS) to reach the specified cfu per larva. The infected larvae were collected in a Petri dish and incubated at 37°C. The viability of the larvae was assessed twice a day up to a total of 72 hours post-infection by checking for movement. Larvae were considered dead if no movement could be observed in response to stimulus with a pipette tip.

### Quantitative reverse transcription-PCR

Quantitative reverse transcription-PCR (qRT-PCR) was performed as previously described [40]. Briefly, *A. baumannii* isolates were grown in LB broth at 37°C to mid-log phase (OD600 = 0.5), and total RNA was extracted using a PureLink RNA minikit (Ambion) according to the manufacturer’s recommendations. When compound treatment was performed, *A. baumannii* isolates were grown to an OD600 of 0.3 and incubated with the specified compound concentration for 30 min before total RNA was extracted as mentioned above. qRT-PCR was performed using a GoTaq 1-Step RT-qPCR System kit (Promega) and expression levels were normalized to that of the housekeeping gene *rpoD* using the comparative threshold cycle (ΔΔCT) method. A detailed description of the DNA probes used for RNA detection is given in Table S4.

### RNA sequencing (RNAseq)

Total RNA samples of *A. baumannii* AB5075 and its Δ*ompR* mutant were prepared in triplicate following the method mentioned above for qRT-PCR. RNA libraries were prepared with an Illumina TrueSeq (including rRNA depletion) and sequenced with Illumina NextSeq (1x75-bp) at Microsynth AG (Balgach, Switzerland). The reads were mapped to *A. baumannii* AB5075 genome (NZ_CP008706) using TopHat (v 2.1.1), mapped reads were counted using HTSeq (v 0.6.0) and statistical analysis was performed using DESeq2 (v 1.6.3).

### Protein production

A DNA fragment encoding *A. baumannii* OmpR full-length (OmpR-FL) (Uniprot ID: B0VPC9) without the first eighteen *N*-terminal residues was purchased as a codon optimized gene for *E. coli* expression from Genewiz. A DNA fragment encoding the C-terminal DNA-binding domain of OmpR (OmpR-DBD, amino acid 148 to 254) was amplified by PCR using the synthetic gene as a template and both OmpR-FL and OmpR-DBD DNA fragments were cloned into a modified version of the pET15 expression plasmid [57]. The DNA fragment encoding OmpR R198L was amplified by PCR from *A. baumannii* genomic DNA and cloned in the pET28a expression plasmid.

Bacteria harbouring the expression plasmids were grown to OD600 = 0.6 in LB medium containing the appropriate antibiotic and protein expression was induced with 1 mM IPTG at 18 °C for 18 hours for OmpR-FL and OmpR-DBD or 30°C for 6 hours for OmpR R198L. After cell lysis, the proteins were purified on a HisTrap column and the fractions containing the target proteins were pooled. The OmpR-FL and OmpR-DBD proteins were further purified on a HiLoad 16/60 Superdex 75 prep-grade column using gel filtration buffer (50 mM Tris pH 7.5, 200 mM NaCl). The OmpR R198L protein was dialysed overnight a 4°C in the dialysis buffer (20 mM Tris/HCl pH 7.5, 100 mM NaCl, 25% glycerol). Proteins were flash-frozen in liquid nitrogen and stored in aliquots at −80°C.

### Crystallization of DNA binding domain of OmpR

Protein crystals were obtained using sitting-drop vapour diffusion method. Briefly, the OmpR-DBD protein was mixed in a solution consisting of 0.1 M BisTris propane (pH 8.5), 0.2 M Na_2_SO_4_ and 22% PEG 3350 and incubated at room temperature for 1 week to enable crystal growth. Diffraction data were collected on beamline PXIII at PSI, Villigen, Switzerland at 100 K. Data reduction and processing was carried out using XDS, programs from the CCP4 suite (Collaborative Computational Project 4, 1994). The crystal structure was solved via molecular replacement method using the crystal structure of DBD of OmpR from *E. coli* as a template (PDB code: 1OPC) [31]. Refmac was used for refinement and Coot was used for manual building of the model [58],^59^]. Data and refinement statistics are shown in (Supplementary Table S5). All structure figures were prepared using PyMOL (Schrödinger, LLC, New York). The structure was deposited in the protein data bank with accession code 6ZWT.

### Hot-spot analysis

Hot-spot analysis and prediction of potential ligand-binding sites was performed by inSili.com LLC (inSili.com). An in-house software tool for hot-spot analysis was used for identifying potential protein-ligand binding sites on the solvent-accessible surface of protein structures 1OPC and 6ZWT (from PDB). The software captures potential protein–ligand interaction points, including protein–protein interactions. It is based on a machine-learning classifier trained to the recognition of known protein–ligand interaction sites (surface patches). Protein monomers or individual chains were analysed.

### DNA binding enzyme-linked immunosorbent (D-ELISA) assay

The D-ELISA assay was used to study OmpR full length protein-DNA binding. Clear flat bottom high-binding capacity polystyrene 96-well plates (Costar™ 3590, 96-Well EIA/RIA Plates) were coated overnight at 4°C with 100 µl streptavidin (4 µg/ml) and blocked for 2 hours at room temperature using blocking buffer (10 mM Na_2_HPO_4_, 10 mM NaH_2_PO_4_, 10 mM NaCl, 0.05% of Tween 20 and 1% milk powder at pH 7). The plates were washed using the washing buffer (blocking buffer without Tween 20 and milk powder) and 100 µl of annealed biotinylated oligonucleotides diluted in blocking buffer were added to the plates for 1 hour. After washing, 100 µl of the protein diluted in blocking buffer were added and the plates were incubated for 1 hour. The plates were washed and protein-DNA binding was detected using 100 µl of monoclonal mouse antibody anti-6X His HRP (Rockland, 200-303-382) diluted to 100 ng/ml in blocking buffer for 1.5 hours followed by the addition of the TMB substrate (SouthernBiotech, 0410–01). The reaction was stopped after 30 min with 50 µl of 0.5 M H_2_SO_4_ (Sigma Aldrich, 320501) and the resulting absorbance was determined at 450 nm with the TECAN infinite F500. When specified, protein phosphorylation was done in the presence of 150 mM lithium acetyl phosphate at 30°C for 30 min. The proteins were pre-incubated (30 min) with the specified compound concentration to assess protein-DNA binding inhibition.

### Phos-tag gel

The phosphorylation state of OmpR was evaluated using 13% SDS-PAGE gels supplemented with Phos-tag^TM^, according to the manufacturer recommendation. The gels were run at 150 V and then fixed in a solution containing 40% methanol and 10% acetic acid before staining with Coomassie Brilliant Blue (0.25%).

### Swarming motility assay

Swarming motility was assessed as previously described [60]. Briefly, the inoculum was prepared by resuspending 4–6 freshly growing colonies in 100 µl LB, and 2 µl were spotted in the middle of a freshly prepared Nutrient broth agar (0.5% Eiken agar) plate. The plates were incubated in upright position at 35°C and the swarming diameter was measured after 48 hours.

### Antibacterial activity and cytotoxicity assays

The antibacterial activity of VSIS_039 was determined in microbroth dilution MIC according to CLSI guidelines. Briefly, bacterial inoculum was prepared at 5 × 10^5^ cfu/mL in cation adjusted Mueller Hinton broth (CA-MHB). Two-fold dilution series of the compound were prepared in CA-MHB at 10-fold the final concentration and 10 µl compound was mixed with 90 µl of bacterial inoculum in a 96-well assay plate. The plate was incubated at 35°C for 20 hours and the MIC was read as the lowest concentration that inhibited visual growth.

The cytotoxicity of VSIS_039 was determined using HepG2 cell line engineered for stable expression of the human secreted embryonic alkaline phosphatase (hSEAP) [61]. HepG2-hSEAP cultured in Minimum Essential Medium Eagle supplemented with 10% foetal bovine serum were seeded at 2 × 10^4^ cells/well in a tissue culture graded 96-well plate and incubated at 37°C (5% C0_2_). After 24 hours, the medium was replaced by fresh medium containing 2-fold dilution of the compound. The cells were incubated for 24 hours with the compound and cell viability was assessed by hSEAP expression quantification. Briefly, heat inactivated cell culture supernatant was mixed with *p*-nitrophenyl phosphate at 12 mM in 2x SEAP buffer (20 mM homoarginine, 1 mM MgCl_2_, 21% diethanolamine, pH 9.8) and the absorbance was read at 405 nm for 15 min. Half-lethal dose (LD_50_) was defined as the lowest concentration that inhibited more than 50% hSEAP expression compared to untreated control.

## Supplementary Material

Supplemental Material

## Data Availability

All data are fully available without restriction.
