# Supplementary material for: Targeting virulence regulation to disarm Acinetobacter baumannii pathogenesis
Source: Virulence. 2022 Oct 19;13(1):1868–83. doi: 10.1080/21505594.2022.2135273 (PMC9586577; doi:10.1080/21505594.2022.2135273)
Supplement: Supplemental Material [file KVIR_A_2135273_SM7587.pdf]

Supplementary data

Targeting virulence regulation to disarm *Acinetobacter baumannii* pathogenesis

Vincent Trebosc[1,a], Valentina Lucchini[1,a,b], Mohit Narwal[1,c], Basil Wicki[a], Sarah Gartenmann[a], Birgit Schellhorn[a], Julian Schill[a], Marilyne Bourotte[c], Daniel Frey[d], Jürgen Grünberg[e], Andrej Trauner[a], Livia Ferrari[f], Antonio Felici[f], Olivia L. Champion[g], Marc Gitzinger[a], Sergio Lociuro[a], Richard A. Kammerer[c], Christian Kemmer[a], Michel Pieren[*a]

[a] BioVersys AG, Basel, Switzerland

[b] Biozentrum, University of Basel, Basel, Switzerland

[c] BioVersys SAS, Lille, France

[d] Laboratory of Biomolecular Research, Division of Biology and Chemistry, Paul Scherrer Institute, Villigen, Switzerland

[e] Center for Radiopharmaceutical Sciences ETH-PSI-USZ, Paul Scherrer Institute, Villigen, Switzerland

[f] Aptuit Srl, an Evotec Company, Verona, Italy

[g] Biosystems Technology Ltd, Exeter, UK

[1] Contributed equally

[*] Corresponding author: Michel Pieren, michel.pieren@bioversys.com

Supplementary Figures

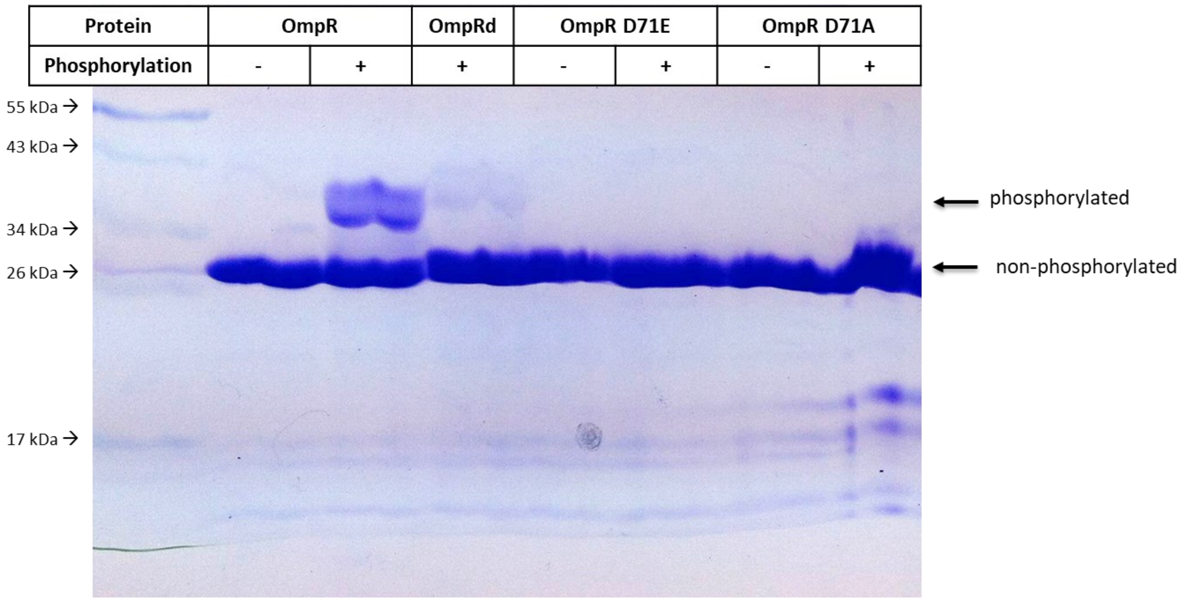

Figure S1: Phosphorylation of OmpR variants.

The OmpR variants from *A. baumannii* were phosphorylated *in vitro* and the phosphorylation was analyzed used Phos-tag SDS gel. Protein retention is significative of protein phosphorylation. Denatured OmpR (OmpRd, 5 min at 95°C) was used as control.

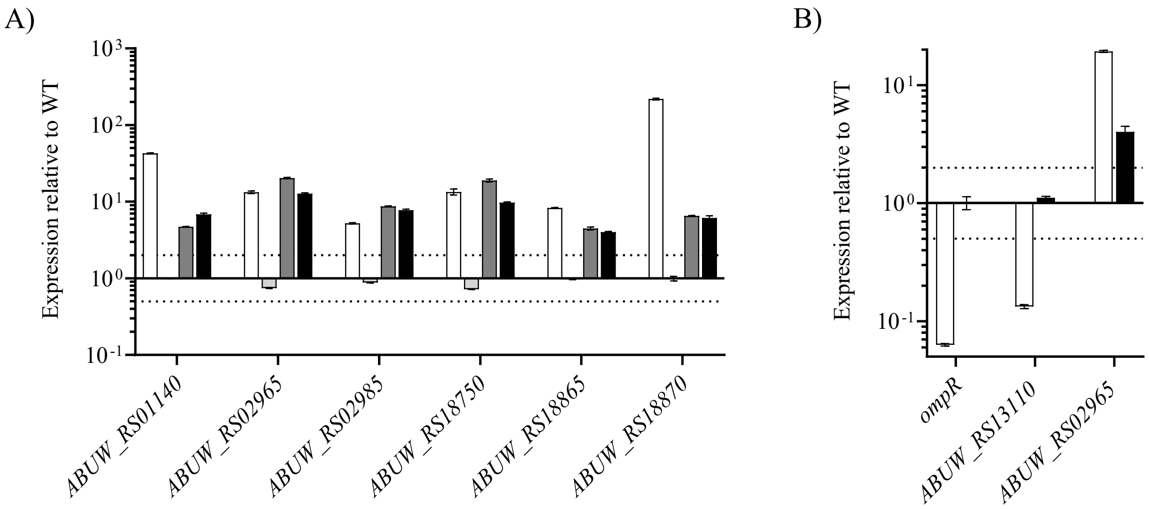

Figure S2: Confirmation of OmpR regulated genes using qRT-PCR on the chromosomal mutants and the complemented strain.

A) The transcript levels of OmpR upregulated genes encoding small hypothetical protein were determined in *A. baumannii* AB5075 wildtype, AB5075 Δ*ompR* (white), AB5075 OmpR D71E (light grey), AB5075 OmpR D71A (dark grey) and AB5075 OmpR R198L (black) mutants by quantitative real-time PCR and the expression level was normalized to the expression of the AB5075 wildtype strain (means ± SEM of 2 technical replicates). B) The transcript levels of *ompR*, *ABUW_RS13110* and *ABUW_RS02965* were determined in *A. baumannii* AB5075 wildtype, AB5075 Δ*ompR* (white) and AB5075 OmpR complemented (black) strains by quantitative real-time PCR and the expression level was normalized to the expression of the AB5075 wildtype strain (means ± SEM of 2 technical replicates). Horizontal dotted lines depict a 2-fold up- or downregulation.

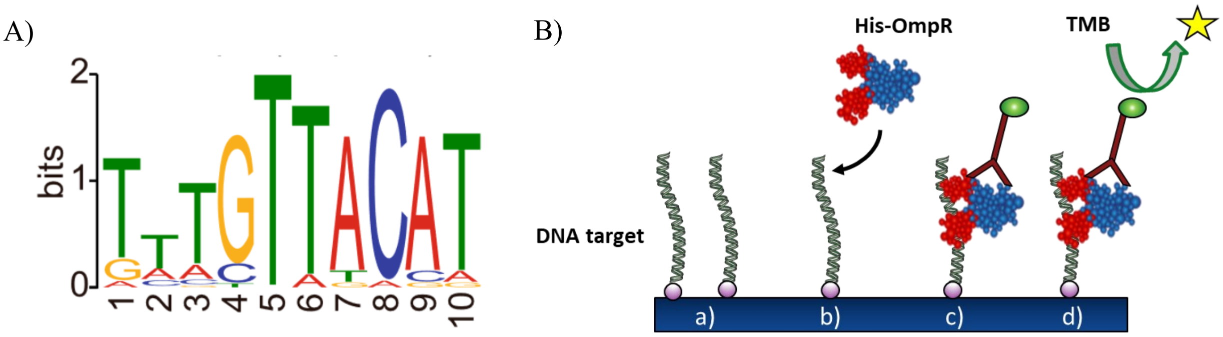

Figure S3: OmpR DNA binding motif and method to study OmpR-DNA binding *in vitro*.

(A) *E. coli* OmpR DNA binding Weblogo determined by Seo et al [18] (B) DNA binding enzyme-linked immunosorbent (D-ELISA) sandwich method to quantify OmpR-DNA binding: a) coating of D-ELISA plate with streptavidin followed by immobilization of biotinylated oligonucleotides, b) OmpR His-tag protein binding to immobilized oligonucleotides, c) anti-His-HRP antibody binding to bound OmpR and d) HRP-based enzymatic quantification of bound OmpR.

ABUW_RS02965 AGCAATTGACCTAGCTTGAAAAATGCTAA**TGGTAGCACA**GATACAGATTCTTCATATTTTCTTTATAGTCATTACAAACAATATCAATCAAAGCCATTTTATTCATGGTTTCTCCTTGACTCTCTCTTAATCTAGCTTCAGATCAAGATAAAAAGGAATACTCCATG

ABUW_RS02965_A TTGAAAAATGCTAA**TGGTAGCACA**GATACAGATTCTTCATATTTT

ABUW_RS02965_B TTTTCTTTATAGTCATTACAAACAATATCAATCAAAGCCATTTT

ABUW_RS02965_C TTTTATTCATGGTTTCTCCTTGACTCTCTCTTAATCTAGCTTCAGATCAAGAT

ABUW_RS07785 TAGGTCGATTTAGTACAAAAAAAGGAGCTGATTATTCAGCTCCTTTTTATTCTAATGAGAA**TTGGTACATA**TTTTAATTGTTAAAATAATAAATTAATCATGTTTTATTCTAATAAAATGTAAAAATAAATATAACGCTAGATATTAAAGAAGATTTTGTTTTAAAA
TATTTGTATGAACTTAAAAATAATTAAAAAGAACATGGGTATTTTATATATGGAATTTAAAAATG

ABUW_RS02985 AAGCACTCTCTCATTTTTACTCTATATTTTGC**ATTGTAACCA**ACAACGAAAAGAAAAACTCTCCATTGTTTCTTCATCTTTATTATCTAATCTTTAAGTCATCTTAAAACAAACATTAAAGGGTTTTAACCATG

ABUW_RS13110 ATAAATTAAGTCTATTTTTAACTTAAATTTGGTTTTTTTAAAATTTATGTGACGTATTTTGCAAATTTAAAATTGAATAATTTAT**AGTGAAGCTT**GAGTTCATTTTTTATTAATGATTTGATTTTAAAAAATAAAAAATATTTGTAATATTTCTTGTTACAACGATTTT
GTCGACAATACAGGATTGTTTTTGACATATTTCGCCTAGCGTAATCAATAGAGTTTTTTGCCTATG

Figure S4: Sequence of the intergenic region upstream of the OmpR-regulated genes.

The intergenic region upstream of *ABUW_RS02965* has been divided in three to design the oligo ABUW_RS02965_A, ABUW_RS02965_B and ABUW_RS02965_C for D-ELISA assay with $OmpR_{ABA}$ (Figure 4E). The start codon is underlined and the OmpR DNA binding sequence motifs identified with the MEME suite are highlighted in red (Figure 4D). The motifs for ABUW_RS02985 and ABUW_RS13110 were identified on the minus strand.

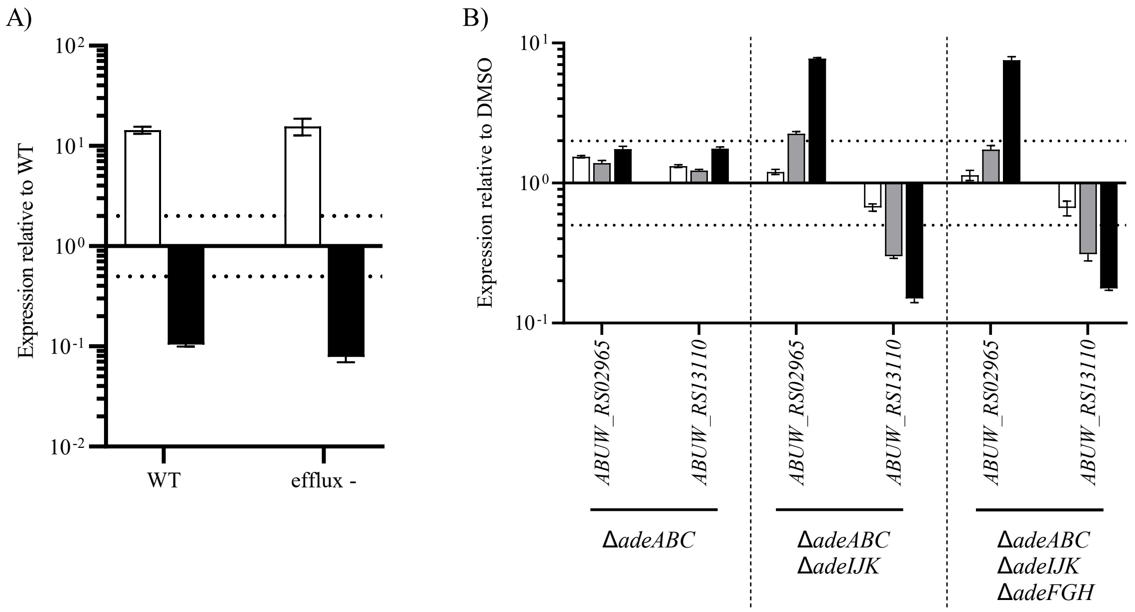

Figure S5: OmpR-regulated genes in the AYE efflux depleted mutant using qRT-PCR.

A) The transcript levels of *ABUW_RS02965* (white) and *ABUW_RS13110* (black) were determined in the Δ*ompR* mutants of the *A. baumannii* AYE wildtype and efflux depleted (Δ*adeABC* Δ*adeFGH* Δ*adeIJK*) mutant and normalized to the transcript levels of their respective wildtype strains (mean ± SEM of 2 technical replicates). B) The transcript levels of *ABUW_RS02965* and *ABUW_RS13110* were determined in the single (Δ*adeABC*), double (Δ*adeABC* Δ*adeIJK*) and triple (Δ*adeABC* Δ*adeFGH* Δ*adeIJK*) efflux deletion mutant of *A. baumannii* AYE in the presence of VSIS_039 at 20 µM (white), 40 µM (grey) and 80 µM (black). The transcripts levels were normalized to those of the DMSO control (mean ± SEM of 2 technical replicates). Horizontal dotted lines depict a 2-fold up- or downregulation.

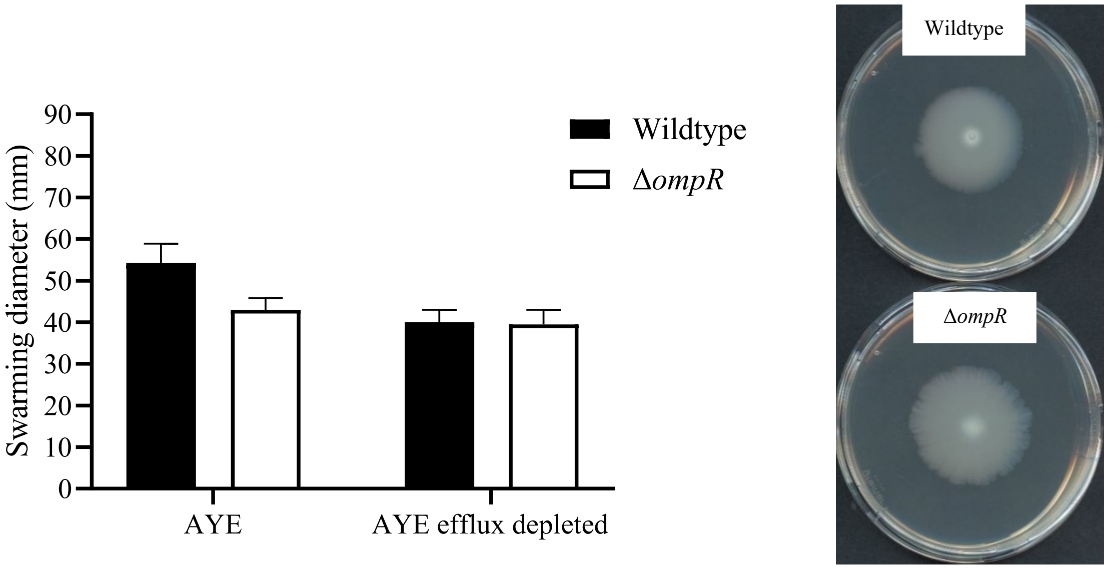

Figure S6: Swarming motility of *A. baumannii* AYE wildtype and *ompR* deleted (Δ*ompR*) strains. Swarming motility was evaluated for the wildtype / Δ*ompR* pair of strains of AYE and AYE efflux depleted strain (Δ*adeABC*Δ*adeIJK*Δ*adeFGH*). The graph reports the swarming diameter while the picture illustrates the swarming of the *A. baumannii* efflux depleted AYE wildtype (up) and Δ*ompR* (down) strains (mean ± SEM of at least 2 replicates).

Supplementary Tables

Table S1: List of the 65 downregulated genes in Δ*ompR* (<-2 log2 fold change, ΔompR/WT) and 71 upregulated genes in Δ*ompR* (>2 log2 fold change, ΔompR/WT).

ID	log2FoldChange (Δ*ompR*/WT)	Product
**ABUW_RS08030**	-5.152963056	TetR family transcriptional regulator
**ABUW_RS11870**	-4.368892816	hypothetical protein
**ABUW_RS12725**	-4.305329643	hypothetical protein
**ABUW_RS07965**	-4.24527092	pilus assembly protein
**ABUW_RS07960**	-3.949138706	hypothetical protein
**ABUW_RS18635**	-3.79767744	UDP-N-acetyl-D-mannosamine dehydrogenase
**ABUW_RS07145**	-3.705203924	hypothetical protein
**ABUW_RS07970**	-3.698264692	fimbrial protein
**ABUW_RS01495**	-3.552227695	prepilin-type cleavage/methylation domain-containing protein
**ABUW_RS13690**	-3.354991113	TetR family transcriptional regulator
**ABUW_RS03355**	-3.354416154	hybrid sensor histidine kinase/response regulator
**ABUW_RS11875**	-3.227852494	hypothetical protein
**ABUW_RS01430**	-3.199573033	membrane protein
**ABUW_RS09450**	-3.191789235	ribose-phosphate pyrophosphokinase
**ABUW_RS13040**	-3.179126428	hypothetical protein
**ABUW_RS10420**	-3.144814662	hypothetical protein
**ABUW_RS15455**	-3.03176635	hypothetical protein
**ABUW_RS13110**	-3.01221703	transporter MatE
**ABUW_RS09455**	-2.98832666	nicotinate phosphoribosyltransferase
**ABUW_RS07975**	-2.905653226	hypothetical protein
**ABUW_RS07785**	-2.888583284	hypothetical protein
**ABUW_RS13815**	-2.887412345	hypothetical protein
**ABUW_RS18630**	-2.835136126	polysaccharide biosynthesis protein
**ABUW_RS07255**	-2.828869248	protein CsuA
**ABUW_RS03350**	-2.808936997	chemotaxis protein
**ABUW_RS01440**	-2.792972915	hypothetical protein
**ABUW_RS06270**	-2.743514067	hypothetical protein
**ABUW_RS11790**	-2.725242202	amino acid ABC transporter permease
**ABUW_RS01290**	-2.703419778	hypothetical protein
**ABUW_RS03345**	-2.667668241	twitching motility protein PilT
**ABUW_RS07800**	-2.661221571	membrane protein
**ABUW_RS01435**	-2.650341865	hypothetical protein
**ABUW_RS14740**	-2.573108743	hemerythrin
**ABUW_RS03335**	-2.45451199	response regulator
**ABUW_RS17270**	-2.451241573	type IV-A pilus assembly ATPase PilB
**ABUW_RS17275**	-2.448651293	type II secretion system protein F
**ABUW_RS00895**	-2.447969798	hypothetical protein
**ABUW_RS06590**	-2.431786046	type II secretion system protein E
**ABUW_RS09960**	-2.390032425	hypothetical protein
**ABUW_RS07250**	-2.386735092	protein CsuA/B

**ABUW_RS08615**	-2.384859643	hypothetical protein
**ABUW_RS03360**	-2.348787548	hypothetical protein
**ABUW_RS00795**	-2.347192551	MBL fold metallo-hydrolase
**ABUW_RS16790**	-2.287666781	hypothetical protein
**ABUW_RS01465**	-2.282931643	glutamate synthase large subunit
**ABUW_RS02875**	-2.272260195	two-component sensor histidine kinase
**ABUW_RS01550**	-2.26632454	pilus assembly protein PilW
**ABUW_RS14710**	-2.262323809	twitching motility protein PilT
**ABUW_RS01425**	-2.231965395	pilus assembly protein PilM
**ABUW_RS03365**	-2.21954724	coproporphyrinogen III oxidase
**ABUW_RS14715**	-2.211650472	twitching motility protein PilT
**ABUW_RS05525**	-2.208639151	hypothetical protein
**ABUW_RS11235**	-2.195499967	ferrous iron transporter B
**ABUW_RS13105**	-2.191786023	hypothetical protein
**ABUW_RS09530**	-2.181595516	TetR family transcriptional regulator
**ABUW_RS01555**	-2.167614921	hypothetical protein
**ABUW_RS18625**	-2.16215534	hypothetical protein
**ABUW_RS18640**	-2.161371559	UDP-N-acetylglucosamine 2-epimerase (non-hydrolyzing)
**ABUW_RS17370**	-2.157315431	peptidoglycan hydrolase
**ABUW_RS13035**	-2.115209735	hypothetical protein
**ABUW_RS11850**	-2.095210977	NAD(P)-dependent oxidoreductase
**ABUW_RS01445**	-2.069233992	membrane protein
**ABUW_RS07980**	-2.041806136	glutathione S-transferase
**ABUW_RS11425**	-2.008071889	tautomerase
**ABUW_RS01275**	-2.00763149	fatty acyl-CoA reductase
**ABUW_RS15165**	2.002104113	trehalose-6-phosphate synthase
**ABUW_RS04980**	2.005017416	hypothetical protein
**ABUW_RS11915**	2.006015486	SAM-dependent methyltransferase
**ABUW_RS07905**	2.02022884	ethanolamine permease
**ABUW_RS08920**	2.044784618	3-oxoadipyl-CoA thiolase
**ABUW_RS11925**	2.052534622	TetR family transcriptional regulator
**ABUW_RS14335**	2.07063006	(2Fe-2S)-binding protein
**ABUW_RS12645**	2.07683365	acetyl/propionyl-CoA carboxylase subunit alpha
**ABUW_RS09050**	2.110067963	hypothetical protein
**ABUW_RS07625**	2.111922975	luciferase family oxidoreductase
**ABUW_RS03655**	2.114704215	hypothetical protein
**ABUW_RS03690**	2.115251333	DNA replication protein DnaD
**ABUW_RS11950**	2.143126695	hydroxymethylglutaryl-CoA lyase
**ABUW_RS09360**	2.149858781	hydrolase
**ABUW_RS03205**	2.152831275	RNA-splicing ligase RtcB
**ABUW_RS05450**	2.154458373	aromatic amino acid transporter AroP
**ABUW_RS13610**	2.180757921	gamma-aminobutyraldehyde dehydrogenase
**ABUW_RS09260**	2.197897889	sorbosone dehydrogenase
**ABUW_RS09060**	2.204598518	(2Fe-2S)-binding protein
**ABUW_RS08205**	2.215393814	lactonase
**ABUW_RS11920**	2.231187048	fatty acid--CoA ligase
**ABUW_RS02945**	2.251007273	hypothetical protein

**ABUW_RS11940**	2.253834891	enoyl-CoA hydratase
**ABUW_RS15140**	2.27409912	malonyl-[acyl-carrier protein] O-methyltransferase BioC
**ABUW_RS09065**	2.275172651	aromatic-ring-hydroxylating dioxygenase subunit beta
**ABUW_RS11945**	2.275395335	3-methylcrotonyl-CoA carboxylase subunit alpha
**ABUW_RS08915**	2.310124094	3-oxoadipate CoA-transferase subunit B
**ABUW_RS12665**	2.319789099	hypothetical protein
**ABUW_RS12650**	2.406656548	allophanate hydrolase
**ABUW_RS11935**	2.416654319	methylcrotonoyl-CoA carboxylase
**ABUW_RS15145**	2.425254961	8-amino-7-oxononanoate synthase
**ABUW_RS17915**	2.42534756	ATPase
**ABUW_RS12660**	2.454310317	hypothetical protein
**ABUW_RS14330**	2.470163045	hypothetical protein
**ABUW_RS03685**	2.504196589	hypothetical protein
**ABUW_RS15150**	2.525208161	adenosylmethionine--8-amino-7-oxononanoate aminotransferase BioA
**ABUW_RS11930**	2.528314598	isovaleryl-CoA dehydrogenase
**ABUW_RS19690**	2.549252604	hypothetical protein
**ABUW_RS00325**	2.5730652	4-hydroxyphenylpyruvate dioxygenase
**ABUW_RS08910**	2.575615162	3-oxoadipate CoA-transferase subunit A
**ABUW_RS04555**	2.616247733	ABC transporter substrate-binding protein
**ABUW_RS08055**	2.625805749	hypothetical protein
**ABUW_RS01745**	2.628652304	hypothetical protein
**ABUW_RS07295**	2.675155384	proline/betaine transporter
**ABUW_RS05445**	2.689329246	pyruvate decarboxylase
**ABUW_RS12655**	2.695847506	DUF1445 domain-containing protein
**ABUW_RS11470**	2.707164847	glutamate symport protein
**ABUW_RS18865**	2.739894706	hypothetical protein
**ABUW_RS09045**	2.743885166	acyl-CoA dehydrogenase
**ABUW_RS07640**	2.825507594	hypothetical protein
**ABUW_RS13855**	2.889418797	amino-acid transporter Mb2008
**ABUW_RS09040**	2.903434927	Glu-tRNA amidotransferase
**ABUW_RS07910**	2.972202959	aldehyde dehydrogenase
**ABUW_RS10360**	3.120148412	lipoyl synthase
**ABUW_RS10340**	3.331455667	dihydrolipoyl dehydrogenase
**ABUW_RS00825**	3.394383298	outer membrane protein
**ABUW_RS15475**	3.503486865	hypothetical protein
**ABUW_RS02985**	3.673087414	hypothetical protein
**ABUW_RS01270**	3.746149042	sulfate permease
**ABUW_RS06025**	3.783910616	membrane protein
**ABUW_RS07925**	4.027868667	alcohol dehydrogenase
**ABUW_RS07950**	4.186229554	hypothetical protein
**ABUW_RS18750**	4.279546979	hypothetical protein
**ABUW_RS10345**	4.313013618	diaminohydroxyphosphoribosylaminopyrimidine deaminase
**ABUW_RS07630**	4.328430911	hypothetical protein
**ABUW_RS01140**	4.375161033	hypothetical protein
**ABUW_RS10355**	4.750588843	ABC transporter substrate-binding protein
**ABUW_RS10350**	4.809402495	pyruvate dehydrogenase subunit beta

**ABUW_RS02965**	5.333078805	hypothetical protein
**ABUW_RS14395**	5.540563984	hypothetical protein
**ABUW_RS18870**	6.084762391	hypothetical protein

Table S2: List of the 19 OmpR-activated candidate genes and 12 OmpR-repressed candidate genes potentially relevant for *A. baumannii* pathogenesis.

ID	log2FoldChange (Δ*ompR*/WT)	Read Ratio (*G. mellonella*/LB)[1]	Product
**ABUW_RS08030**	-5.152963056	0.043739863	TetR family transcriptional regulator
**ABUW_RS11870**	-4.368892816	0.058780238	hypothetical protein
**ABUW_RS07965**	-4.24527092	0.052994378	pilus assembly protein
**ABUW_RS18635**	-3.79767744	0.053481349	UDP-N-acetyl-D-mannosamine dehydrogenase
**ABUW_RS07145**	-3.705203924	0.054538365	hypothetical protein
**ABUW_RS07970**	-3.698264692	0.139174375	fimbrial protein
**ABUW_RS11875**	-3.227852494	0.195565828	hypothetical protein
**ABUW_RS13040**	-3.179126428	0.184394736	hypothetical protein
**ABUW_RS13110**	-3.01221703	0.141217409	transporter MatE
**ABUW_RS07975**	-2.905653226	0.120654172	hypothetical protein
**ABUW_RS07785**	-2.888583284	0	hypothetical protein
**ABUW_RS09960**	-2.390032425	0.132031752	hypothetical protein
**ABUW_RS03365**	-2.21954724	0.190465744	coproporphyrinogen III oxidase
**ABUW_RS11235**	-2.195499967	0.158606732	ferrous iron transporter B
**ABUW_RS09530**	-2.181595516	0.14107257	TetR family transcriptional regulator
**ABUW_RS18625**	-2.16215534	0.08692852	hypothetical protein
**ABUW_RS13035**	-2.115209735	0.004379322	hypothetical protein
**ABUW_RS11850**	-2.095210977	0.121539888	NAD(P)-dependent oxidoreductase
**ABUW_RS11425**	-2.008071889	0.008644153	tautomerase
**ABUW_RS04980**	2.005017416	1.085639579	hypothetical protein
**ABUW_RS11925**	2.052534622	0.934376763	TetR family transcriptional regulator
**ABUW_RS15140**	2.27409912	0.968768335	malonyl-[acyl-carrier protein] O-methyltransferase BioC
**ABUW_RS15150**	2.525208161	1.53242563	adenosylmethionine--8-amino-7-oxononanoate aminotransferase BioA
**ABUW_RS00325**	2.5730652	1.125844462	4-hydroxyphenylpyruvate dioxygenase
**ABUW_RS01745**	2.628652304	1.162884654	hypothetical protein
**ABUW_RS18865**	2.739894706	2.040619566	hypothetical protein
**ABUW_RS02985**	3.673087414	1.582951167	hypothetical protein
**ABUW_RS18750**	4.279546979	1.001278226	hypothetical protein
**ABUW_RS01140**	4.375161033	1.509661043	hypothetical protein
**ABUW_RS02965**	5.333078805	0.991747211	hypothetical protein
**ABUW_RS18870**	6.084762391	1.271610231	hypothetical protein

[1] Values derived from Gebhardt, M. J. et al. 2015 [1]

Table S3: Overview of the *A. baumannii* strains used in this study.

Strain	Description				Reference
	ST	Origin	Source	Year	
AB5075	1	USA	osteomyelitis	2008	[2]
AB5075 Δ*ompR*	*ompR* deletion in the AB5075 strain				this study
AB5075 OmpR complemented	chromosomal insertion of *ompR* in the AB5075 Δ*ompR* strain				this study
AB5075 OmpR D71A	chromosomal mutagenesis to introduce OmpR D71A in AB5075				this study
AB5075 OmpR D71E	chromosomal mutagenesis to introduce OmpR D71E in AB5075				this study
AB5075 OmpR R198L	chromosomal mutagenesis to introduce OmpR R198L in AB5075				this study
AYE	1	France	urinary tract sample	2001	[3]
AYE Δ*ompR*	*ompR* deletion in the AYE strain				this study
AYE Δ*adeABC*	*adeABC* efflux pump deletion in the AYE strain				this study
AYE Δ*adeABC*Δ*adeIJK*	*adeIJK* efflux pump deletion in the AYE Δ*adeABC* strain				this study
AYE Δ*adeABC*Δ*adeIJK*Δ*adeFGH*	*adeFGH* efflux pump deletion in the AYE Δ*adeABC*Δ*adeIJK* strain				this study
HUMC1	2	USA	blood isolate	2009	[4]
HUMC1 Δ*ompR*	*ompR* deletion in the HUMC1 strain				this study
LAC-4	10	USA	clinical outbreak	1997	[5]
LAC-4 Δ*ompR*	*ompR* deletion in the LAC-4 strain				this study
ATCC-17978	77	France	fatal meningitis	1951	[6]
ATCC-17978 Δ*ompR*	*ompR* deletion in the ATCC-17978 strain				this study
BV191	2	China	respiratory endotracheal aspirate	2013	[7]
BV191 Δ*ompR*	*ompR* deletion in the BV191 strain				this study

Table S4: Primers used in this study.

Name	Sequence	Description
**Genetic engineering**		
ompR deletion		
oVT170	tgaggcctctcgaggaattcGCGGATGGCAGCTTCTAC	*ompR*-up F
oVT171	aacaaacacgagctcGATGTACTCCCTTTTAAAGTCATAATTTATATATTATTG	*ompR*-up R
oVT172	gagtacatcgagctcGTGTTTGTTCCGGATGGTG	*ompR*-down F
oVT173	aggatccccgggtaccATTCGTTGGCAGCATTTTG	*ompR*-down R
oVT179	ACCTTTTCACGGCGCTCATA	*ompR* deletion verification F
oVT180	TCCCGCTAGCATGATTCGTC	*ompR* deletion verification R
ompR chromosomal complementation		
oVT425	caggggagaattgaggcctctcgagTATCGTCAAGCTCAGCGC	*ponA*-up F
oVT426	gtaccgagctcgaattcATCTCCAGAACGTCGTATGC	*ponA*-up R
oVT427	ggagatgcctgaattcgagctcggtacccgggactagtggatcctctagagcctgcagCACCTCCAGCG ATGAAAC	*rrm*-down F
oVT428	ctatcaacaggagtccaagagtcgaccatggTGTGGCGAGGGCTATTAC	*rrm*-down R
oVT630	catacgacgttctggagatgaattcTCGTGTTTGATCCCACAAG	*ompR* complementation F
oVT631	tcatcgctggaggtgctgcagGCAACGTAGTCGGTAAAGG	*ompR* complementation R
oVT430	TGCTCCTGAGACAACTGACG	complementation verification F
oVT431	GTGTGAACAGGGCCATAGCT	complementation verification R
ompR allelic exchange		
oVT634	agaattgaggcctctcgaggaattcTGGGCCTTTAAGCTAGCC	*ompR*-D71 F
oVT635	tctagaggatccccgggtaccTTGCGGTGGTATTGGCTC	*ompR*-D71 R
oVT636	CTTATCGTGCTCGAGTTTATGTTGCCCG	*ompR*-D71E mutagenesis F
oVT637	CGGGCAACATAAACTCGAGCACGATAAG	*ompR*-D71E mutagenesis R
oVT638	CTTATCGTGCTAGCCTTTATGTTGCCCG	*ompR*-D71A mutagenesis F
oVT639	CGGGCAACATAAAGGCTAGCACGATAAG	*ompR*-D71A mutagenesis R
oVT642	agaattgaggcctctcgaggaattcTACTGAGGGCTTGTTGGC	*ompR*-R198 F
oVT643	tctagaggatccccgggtaccCAACCCAGCCTACCAGTAG	*ompR*-R198 R
oVT644	CGTGAACCTTTAACTCTAGACAAGCTCATGAAC	*ompR*-R198L mutagenesis F
oVT645	GTTCATGAGCTTGTCTAGAGTTAAAGGTTCACG	*ompR*-R198L mutagenesis R
Efflux pump deletions		
oCK558	ggaattcGTCAGCGTCAGATTAAGCAAG	*adeABC*-up F
oCK559	cccgggCCAAACCTAGTGAGTTTTTGATG	*adeABC*-up R
oCK560	ACTCACTAGGTTTGGcccgggGCTGATAGAGGTATTCCAAATC	*adeABC*-down F
oCK561	gctctagaGCGTGAAGATTACCATGCAGC	*adeABC*-down R
oCK564	GGCCAGCTCATTACTATTCC	*adeABC* deletion verification F
oCK565	GGCATCGTCGACAATAATCC	*adeABC* deletion verification R
oCK584	aaggcctgaattcGGCAAGTACACTTTCAACGG	*adeIJK*-up F
oCK585	cccgggGACATCATTGTTCCACCTCG	*adeIJK*-up R
oCK586	CGAGGTGGAACAATGATGTCCCCGGGTGGTACATCAACCATCTAGTG	*adeIJK*-down F
oCK587	cgggatccggtaccGGCCTAACTGATCACTTGTG	*adeIJK*-down R
oCK590	CAACAGGTGCTAACGCACTC	*adeIJK* deletion verification F
oCK591	AAAGTGCCGGTGTGAACGTC	*adeIJK* deletion verification R
oCK627	agaattgaggcctctcgaggaagaattcAGCGGTTGCACACTGGAAAG	*adeFGH*-up F
oCK628	ttgtcccgggGAGGTGCTCCTAGTTATTTGG	*adeFGH*-up R
oCK629	ggagcacctccccgggACAAATTATTTGCATATACAGGG	*adeFGH*-down F
oCK630	ccgcaagcttcctgcaggctctaGACATCGCGGACAAAGTTGG	*adeFGH*-down R
oCK633	GAAGATGTACAGCGCTTAGG	*adeFGH* deletion verification F
oCK635	AGCTCCTTCATTGTGCTACG	*AdeFGH* deletion verification R
**qRT-PCR**		
oVT572	TGCGATGGCCGATTCAAATC	*ABUW_RS02965* F
oVT573	TCATTGCGGTGGTCATAACG	*ABUW_RS02965* R
oVT683	ATGGGTAGTTTGGCTGCTTG	*ABUW_RS02985* F
oVT684	CACGGTGTTTCATACGATGGTG	*ABUW_RS02985* R
oVT661	TTATCAGCAGAAGACGGGAATAC	*ABUW_RS07785* F
oVT662	CCCAACGCTCAGCATTTAACTC	*ABUW_RS07785* R
oVT582	TGATTCCGGCAGAGGTAATAGG	*ABUW_RS13110* F
oVT583	ATAAAGCCCGCACTCATCAG	*ABUW_RS13110* R
oVT687	TATGGCTGCTTTGGCTGAAC	*ABUW_RS01140* F
oVT688	TCCGCTATTGACCAAATCGC	*ABUW_RS01140* R
oVT697	ATCGCAACAACAGCAATGGC	*ABUW_RS18750* F
oVT698	TGGATGCTGGTATTGATGCG	*ABUW_RS18750* R

oVT681	TAGTGAGTGGGAACGCAAAC	*ABUW_RS18865* F
oVT682	TCACGACGATCCTTTTGCTG	*ABUW_RS18865* R
oVT689	ACAGCTGTTGGTTGTGCATC	*ABUW_RS18870* F
oVT690	ACACCAACACCAGCTTGTAC	*ABUW_RS18870* R
oBV79	GAGTCTAATGGCGGTGGT	*rpoD* F
oBV80	ATTGCTTCATCTGCTGGT	*rpoD* R
**D-ELISA**		
oCK430	CCCCTTTACTTTTGGTTACATATTCCCC	F1aF1b F<br>5' biotinylated
oVT279	GGGGAATATGTAACCAAAAGTAAAGGGG	F1aF1b R
oVL147	TTGAAAAATGCTAATGGTAGCACAGATACAGATTCTTCATATTTT	*ABUW_RS02965 A* F<br>5' biotinylated
oVL148	AAAATATGAAGAATCTGTATCTGTGCTACCATTAGCATTTTTCAA	*ABUW_RS02965 A* R
oVL149	TTTTCTTTATAGTCATTACAAACAATATCAATCAAAGCCATTTT	*ABUW_RS02965 B* F<br>5' biotinylated
oVL150	AAAATGGCTTTGATTGATATTGTTTGTAATGACTATAAAGAAAA	*ABUW_RS02965 B* R
oVL151	TTTTATTCATGGTTTCTCCTTGACTCTCTCTTAATCTAGCTTCAGATCA<br>AGAT	*ABUW_RS02965 C* F<br>5' biotinylated
oVL152	ATCTTGATCTGAAGCTAGATTAAGAGAGAGTCAAGGAGAAACCATGA<br>ATAAAA	*ABUW_RS02965 C* R
-	ATTTGTATTTAATATTTTAACATAAA	SarA binding site F<br>5' biotinylated
-	TTTATGTTAAAATATTAAATACAAAT	SarA binding site R

Table S5. Crystallographic data-collection and refinement statistics

PDB entry	6ZWT
Beamline	SLS (PXIII)
wavelength (Å)	1.00003
no. of images	720
exposure time (s)	0.1
oscillation range (deg)	0.5
transmission (%)	100
Detector	PILATUS 2M
space group	*P1*
a, *b*, *c* (Å)	48.5, 49.7, 52.7
α, β, γ (deg)	90.1, 99.0, 103.9
resolution (Å)	48.2–2.2 (2.3–2.2)
R_{merge} (%)	15.4 (85.3)
$I/\sigma(I)$	6.1 (1.5)
completeness (%)	98.1 (97.3)
Redundancy	3.6 (3.7)
$CC_{1/2}$	98.8 (73.5)
resolution (Å)	48.2–2.2
no. of unique reflections	23437 (1999)
R_{work}, R_{free}	24.2, 27.3
**no. of atoms**	
Protein	3259
other ligands	34
Water	37
***B* factor (Å^2)**	
Protein	35.2
other ligands	47.5
Water	25.1
**root-mean-square deviation**	
bond lengths (Å)	0.009
bond angles (deg)	1.6
**Ramachandran plot (%)**	
residues in most favorable regions	94.6
residues in additional allowed regions	5.2

References

1. Gebhardt MJ, Gallagher LA, Jacobson RK, Usacheva EA, Peterson LR, Zurawski DV, et al. Joint Transcriptional Control of Virulence and Resistance to Antibiotic and Environmental Stress in Acinetobacter baumannii. mBio. 2015;6: e01660-15. doi:10.1128/mBio.01660-15

2. Jacobs AC, Thompson MG, Black CC, Kessler JL, Clark LP, McQueary CN, et al. AB5075, a Highly Virulent Isolate of Acinetobacter baumannii, as a Model Strain for the Evaluation of Pathogenesis and Antimicrobial Treatments. mBio. 2014;5: e01076-14. doi:10.1128/mBio.01076-14

3. Fournier P-E, Vallenet D, Barbe V, Audic S, Ogata H, Poirel L, et al. Comparative genomics of multidrug resistance in Acinetobacter baumannii. PLoS Genet. 2006;2: e7. doi:10.1371/journal.pgen.0020007

4. Bruhn KW, Pantapalangkoor P, Nielsen T, Tan B, Junus J, Hujer KM, et al. Host fate is rapidly determined by innate effector-microbial interactions during Acinetobacter baumannii bacteremia. J Infect Dis. 2015;211: 1296–1305. doi:10.1093/infdis/jiu593

5. Valentine SC, Contreras D, Tan S, Real LJ, Chu S, Xu HH. Phenotypic and Molecular Characterization of Acinetobacter baumannii Clinical Isolates from Nosocomial Outbreaks in Los Angeles County, California. J Clin Microbiol. 2008;46: 2499–2507. doi:10.1128/JCM.00367-08

6. Piechaud M, Second L. [Studies of 26 strains of Moraxella Iwoffi]. Ann Inst Pasteur. 1951;80: 97–99.

7. Trebosc V, Gartenmann S, Royet K, Manfredi P, Tötzl M, Schellhorn B, et al. A Novel Genome-Editing Platform for Drug-Resistant Acinetobacter baumannii Reveals an AdeR-Unrelated Tigecycline Resistance Mechanism. Antimicrob Agents Chemother. 2016;60: 7263–7271. doi:10.1128/AAC.01275-16
